# Anodal Transcranial Direct Current Stimulation (atDCS) of the Primary Motor Cortex (M1) Facilitates Nonconscious Error Correction of Negative Phase Shifts

**DOI:** 10.1155/2022/9419154

**Published:** 2022-05-25

**Authors:** Bettina Pollok, Martin Jurkiewicz, Vanessa Krause

**Affiliations:** ^1^Institute of Clinical Neuroscience and Medical Psychology, Medical Faculty and University Hospital Düsseldorf, Heinrich-Heine University Duesseldorf, 40225 Duesseldorf, Germany; ^2^Department of Neuropsychology, Mauritius Hospital and Neurorehabilitation Center Meerbusch, 40670 Meerbusch, Germany

## Abstract

Accurate motor timing requires the temporally precise coupling between sensory input and motor output including the adjustment of movements with respect to changes in the environment. Such error correction has been related to a cerebello-thalamo-cortical network. At least partially distinct networks for the correction of perceived (i.e., conscious) as compared to nonperceived (i.e., nonconscious) errors have been suggested. While the cerebellum, the premotor, and the prefrontal cortex seem to be involved in conscious error correction, the network subserving nonconscious error correction is less clear. The present study is aimed at investigating the functional contribution of the primary motor cortex (M1) for both types of error correction in the temporal domain. To this end, anodal transcranial direct current stimulation (atDCS) was applied to the left M1 in a group of 18 healthy young volunteers during a resting period of 10 minutes. Sensorimotor synchronization as well as error correction of the right index finger was tested immediately prior to and after atDCS. Sham stimulation served as control condition. To induce error correction, nonconscious and conscious temporal step-changes were interspersed in a sequence of an isochronous auditory pacing signal in either direction (i.e., negative or positive) yielding either shorter or longer intervals. Prior to atDCS, faster error correction in conscious as compared to nonconscious trials was observed replicating previous findings. atDCS facilitated nonconscious error correction, but only in trials with negative step-changes yielding shorter intervals. In contrast to this, neither tapping speed nor synchronization performance with respect to the isochronous pacing signal was significantly modulated by atDCS. The data suggest M1 as part of a network distinctively contributing to the correction of nonconscious negative step-changes going beyond sensorimotor synchronization.

## 1. Introduction

The ability to rapidly adapt one's own movements to changes in the environment allows the flexible and seemingly effortless execution of movements. This is particularly reflected by the elegance of dancers automatically adapting the tempo of their movements with respect to rhythmic changes in a piece of music. Performance monitoring and error correction are a key for the temporally precise adjustment of movements with respect to external pacing signals (for comprehensive overviews, please refer to [1, 2]). Movements can be adapted even with respect to small rhythmic perturbations that were not perceived consciously [3–8] (for review articles, refer to [1, 2]). Such error correction can be investigated by means of the sensorimotor synchronization task that requires the participants to tap in synchrony with an external rhythm (for review articles, refer to [1, 2]). In the easiest version of this task, a regular auditory pacing signal is applied. To induce error correction, local timing perturbations are occasionally interspersed in the regular sequence requiring the participants to adapt subsequent movements [3–9] (for review articles, refer to [1, 2]).

Perceptual as well as motor timing recruits olivo-cerebellar and striato-thalamo-cortical circuits [10, 11]. Motor timing with respect to predictable intervals like a regularly occurring pacing signal (i.e., event-based timing) has been suggested as an automatic process linked to motor and premotor circuits (for a review article, refer to [12]). Timing in the subsecond range has been particularly related to the cerebellum [3, 11, 13, 14] (for review articles, refer to [1, 2, 12, 15, 16]) and the basal ganglia [14] (for review articles, refer to [1, 2, 17]). On the cortical level, evidence for the involvement of the primary sensorimotor cortex (S1/M1) and lateral as well as mesial premotor areas [3, 11, 14, 18–20] (for review articles, refer to [12, 16, 17]) and the inferior parietal cortex exists [3, 14] (for review articles, refer to [15, 17]).

Conscious and nonconscious performance monitoring and error correction may rely on at least partially distinct brain networks [3, 5–7, 21]. Correction of movements with respect to perceivable temporal deviations in rhythmic synchronization (i.e., conscious error correction) has been related to activation changes in the left posterior cerebellar lobe [3, 7], prefrontal [3, 5, 7, 21], and anterior cingulate cortices [5, 21], as well as inferior parietal areas [3, 7]. The brain network underlying correction of nonconscious deviations of the pacing signal is less clear. While evidence for the involvement of bilateral ventral medio-frontal cortices [5] and the dorsal anterior cingulate cortex [6] exists, other studies did not find significant brain activation differences between isochronous tapping and nonconscious error correction [3, 7].

Brain stimulation techniques like transcranial magnetic stimulation (TMS) or transcranial direct current stimulation (tDCS) allow the noninvasive modulation of cortical excitability (for review articles, refer to [22–29]). In tDCS, low constant currents are applied to the brain via electrodes attached to the scalp. Although the exact mechanisms are not entirely understood (for review articles, refer to [24, 30, 31]), evidence exists that tDCS modulates the resting membrane potential of targeted neurons in a polarity-specific manner: While anodal tDCS is assumed to exert a constant depolarization, cathodal tDCS may yield hyperpolarization thereby affecting the excitability of the stimulated area (for review articles, refer to [22, 24–27]). Since stimulation effects are not restricted to the stimulated area due to functional connectivity, tDCS allows the investigation of task-related functional networks (reviewed in [22]). With prolonged stimulation intervals, tDCS effects can outlast the stimulation period [32] (for review articles, refer to [22, 25]).

Previous studies suggest the involvement of M1 in sensorimotor synchronization with respect to an isochronous pacing signal as well as in nonconscious error correction of temporal step-changes [3, 7]. However, neither anodal nor cathodal tDCS applied to M1 yielded a significant effect on synchronization accuracy [33]. We here test the hypothesis whether increasing the M1 excitability by means of anodal tDCS may distinctively facilitate nonconscious error correction. Since previous data do not support the involvement of M1 in conscious error correction [3, 5, 7, 21], we did not expect a stimulation effect on task performance in this condition.

## 2. Material and Methods

### 2.1. Participants

Eighteen healthy volunteers (9 males) aged between 18 and 31 years (23.8 ± 0.5 years; mean ± standard error of the mean (s.e.m.)) participated in the present study. They were not regularly practicing a musical instrument in the last five years prior to their participation. Handedness was assessed using the Edinburgh Handedness Inventory (EHI) [34]. Mean laterality quotient was 96.9 ± 2.1 suggesting that all participants were right-handed. Exclusion criteria were neurological, psychiatric, or internal diseases as well as intake of central nervous system-active medication. Participants with family history of epileptic seizures were also excluded, and we desisted from study participation, when pregnancy was not precluded. The individual health condition was determined by the participants' self-reports. None of them reported motor impairment or impaired hearing abilities. They provided their written informed consent prior to data acquisition. The study was conducted in accordance with the latest version of the Declaration of Helsinki and was approved by the local ethics committee of the medical faculty of the Heinrich-Heine University (study number 3347).

### 2.2. Paradigm

To assess sensorimotor synchronization, an auditory pacing signal (i.e., binaural tone, sine wave, duration 100 ms, and 400 Hz) was presented with a regular interstimulus interval (ISI) of 800 ms via loudspeaker resulting in a stimulus onset asynchrony (SOA) of 900 ms. The ISI was chosen since with intervals between 200 and 1,800 ms healthy participants can reliably predict the pacing signal while with larger intervals they tend to react to it [35]. After 10 regularly presented tones, the ISI randomly changed by either 15% of the regular interval to induce conscious or by 2% to induce nonconscious perturbations (*P*) in a stepwise manner. Step-changes between 10 ms [4, 36] and 18 ms [3] had been used in previous studies to induce nonconscious error correction. Although in those studies, shorter baseline ISIs of either 500 or 600 ms were adopted, it has been shown that the phase correction response does not substantially differ after phase shifts of 2% with baseline intervals ranging between 400 ms and 1,300 ms corresponding to step-changes ranging from 8 to 26 ms (for a review article, refer to [2]).

**Figure 1:**
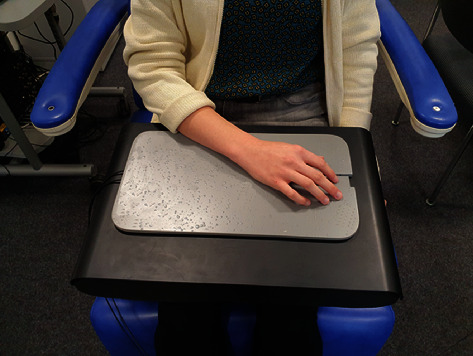
Experimental set-up. The participants were comfortably seated in a reclining chair, and the tapping board was placed on the participants' thigh. Tap-onsets were registered by means of a photoelectric barrier mounted on the tapping board (Elekta Neuromag®, Helsinki, Finland).

In previous studies, conscious error correction was induced by step-changes of either 50 ms [4, 36] or 90 ms [3]. Due to data from a pilot study, suggesting that the threshold for reliably recognizing temporal deviations in a sequence of otherwise regularly presented tones was about 15% (unpublished data), we set the phase shifts for conscious error correction to 120 ms. After each step-change (*P*), the ISI was kept constant for another four taps (*P* + 1, *P* + 2,…,*P* + 4) and then switched back to the initial ISI. Step-changes occurred in either direction (i.e., 816 ms and 784 ms in nonconscious trials and 920 ms and 680 ms in conscious trials) and were repeated three times, respectively. The task took about 3 minutes, and each experimental session lasted for about 45 minutes. The task was realized without breaks and without providing feedback regarding task performance. Timing of the pacing signal as well as registration of tap-onsets was realized by E-Prime (Psychology Software Tools Inc., Sharpsburg, PA, USA). Tap-onsets were measured by a photoelectric barrier mounted on a tapping board (Elekta Neuromag®, Helsinki, Finland) which was placed on a tray on the participants' thigh allowing them to take in a comfortable position (Figure 1). The photoelectric barrier was connected to a converter attached to a standard Windows PC. Please note that the task was purely auditory.

**Figure 2:**
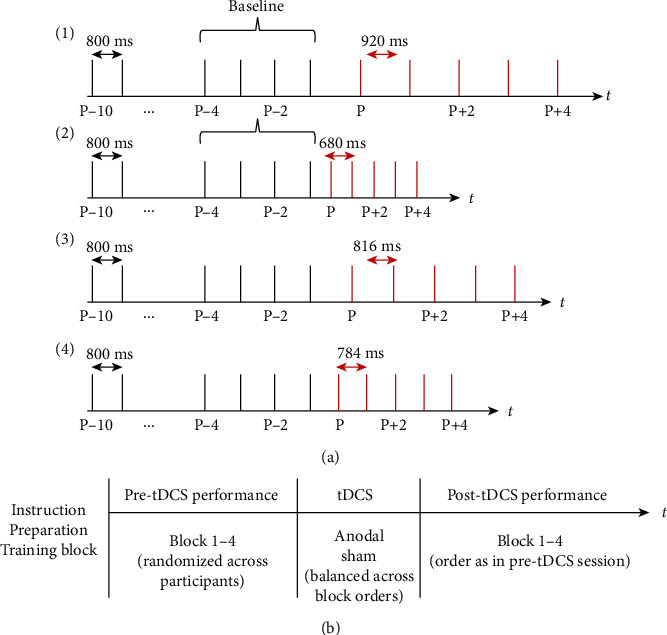
(a) Task and (b) study design. (a) After 10 isochronous auditory pacing signals, a step-change (*P*) was introduced that was either conscious (1, 2) or nonconscious (3, 4). Step-changes were either positive (1, 3) or negative (2, 4). Each block (1–4) was presented three times in a randomized order. The four taps preceding the step-change (*P*) were averaged separately for each experimental condition as a measure of baseline synchronization performance. (b) After providing written informed consent, the participants were prepared for tDCS and performed a training block. Then, prestimulation performance was tested, and subsequently, tDCS was applied to the left M1 either with anodal polarity for 10 minutes or in a sham-condition for 30 seconds. Thereafter, post-tDCS performance was measured, and finally, the type of stimulation as well as possible tDCS-related adverse effects was determined by questionnaire.

The pacing signal was presented via standard speakers. Volume was individually adjusted in a way that the signal was well audible. During the experiment, the participants were comfortably seated in a reclining chair and were instructed to synchronize the taps of their right index finger with respect to the tone-onsets as precisely as possible. They were informed that the pacing signal may contain occasional temporal irregularities. We decided to inform the participants about the irregularities beforehand since data from a pilot study (unpublished data) suggest that uninformed participants tend to omit the tap after the step-change at least in trials with conscious deviations. Prior to each experimental session, a training block was provided to familiarize the participants with the task and the apparatus. To this end, each of the experimental blocks (Figure 2) was presented once. A training criterion was not applied.

### 2.3. Transcranial Direct Current Stimulation (tDCS)

The motor-cortical representation of the right first dorsal interosseus (FDI) muscle was localized by means of single pulse TMS. A standard figure of eight coil (MC-B70) connected to a MagPro stimulator (Mag Venture, Hueckelhoven, Germany) was placed tangentially to the scalp with the handle pointing backwards and laterally at about 45° away from the midline to trigger motor evoked potentials (MEPs). The area evoking the largest motor response was identified as motor hot spot by moving the coil in 0.5 cm steps in anterior, posterior, lateral, and mesial direction across the scalp and marked as target area for stimulation. Saline-soaked sponge electrodes attached to the skin surface were used for tDCS. The active electrode (3 × 3 cm^2^) was placed above the left M1, and the return electrode (5 × 5 cm^2^) above the right eye. A larger return electrode was chosen to minimize the possibility of orbitofrontal costimulation [37] (for a review article, refer to [23]). For electrode fixation, self-adhesive bandages (Coban, 3M Deutschland GmbH, Neuss, Germany) were used. A battery-driven DC-Stimulator Plus (Eldith, NeuroConn, Ilmenau, Germany) was established. The left M1 was stimulated due to the left-hemispheric dominance for skilled movements in right-handed participants (e.g., [38]). Anodal tDCS was applied for 10 minutes with additional fade-in and fade-out periods of 10 seconds each [23, 39]. To this end, the intensity was ramped up for the first 10 seconds of stimulation to the final stimulation intensity and ramped down for the last 10 seconds of stimulation. Since abrupt on- and offsets of the current may induce the perception of phosphenes [38], this approach is aimed at ensuring blinding of the participants regarding the stimulation type. Sham stimulation served as control condition and was applied for 30 seconds with additional 10 seconds of fade-in and fade-out periods yielding the typical sensations associated with tDCS like a slight tingling of the skin [23, 39, 40]. Due to the short stimulation period, effects on neuronal excitability exceeding the stimulation period can be widely excluded [23, 39, 40]. Stimulation intensity was set to 250 *μ*A corresponding to current densities of 0.028 mA/cm^2^ below the active electrode and 0.01 mA/cm^2^ below the return electrode. A current density of approximately 0.028 mA/cm^2^ has been shown to significantly modulate M1 excitability, while no significant effects occurred with stimulation intensities below 0.01 mA/cm^2^ [26]. Impedance was kept below 10 k*Ω* and was on average 7.3 ± 0.4 kΩ (anodal) and 7.8 ± 0.4 kΩ (sham; *t*(17) = –1.136, *p* = 0.272). The DC-stimulator switched off automatically in each condition. To control for sufficient blinding of the participants regarding the stimulation condition, they were asked to estimate the stimulation type after each experimental session by questionnaire. To this end, we asked them whether they had received anodal or sham tDCS. In case the participants were uncertain, we asked them to make a guess. In addition, possible stimulation-related adverse effects were determined after each session by a modified questionnaire according to Antal and coworkers [41]. We asked the participants whether they had recognized any changes during or after the stimulation like tingling, burning, pain, itching, or anything else that might be related to the stimulation. In case the participants reported a conspicuous feature, we asked for the intensity (mild vs. intense). We applied tDCS with respect to safety [41, 42] and technical guidelines [23, 39, 43].

### 2.4. Design

A sham-controlled, double-blind within-subject design was applied in the present study. The participants were naïve regarding the exact purpose of the study and the respective stimulation condition. None of them had received electrical brain stimulation before. Blinding of the main investigator regarding the stimulation condition was achieved by a second investigator being responsible for handling the DC-stimulator. The time of day of participation was held constant across experimental sessions for each individual.

The study was conducted in a quiet experimental room, and the participants were asked to keep their eyes open during the entire experiment. After obtaining written informed consent for study participation, the motor-cortical representation of the right FDI was determined by means of single-pulse TMS. Then, the skin covering the target area as well as the area above the right eye was degreased by means of 80% ethanol for subsequent tDCS, and both electrodes were attached to the head. After the training session, prestimulation data were acquired. Subsequently, tDCS was applied, and immediately after this, the behavioral data were again determined. Finally, the stimulation questionnaire was completed. Each participant received anodal and sham tDCS in consecutive sessions. To minimize carry-over effects of the stimulation, sessions were separated by at least one week. The order of tDCS conditions was balanced across participants and block orders. The procedure is summarized in Figure 2.

### 2.5. Data Analysis

Synchronization accuracy was calculated as the temporal distance between tap- and tone-onsets as well as the corresponding variability. To this end, the result files generated by E-Prime were exported to Excel and used for the calculation of the temporal distance of each tap-to-tone pair, as well as individual and group means and the respective standard deviations for each tap-position of interest. Data of two standard deviations above and below individual and group means at each position (*P*–4, *P*–3,…,*P*, *P* + 1,…,*P* + 4) were classified as outliers and discarded from further analysis. On average, 5.4% of all trials were removed from the analysis. Prior to sham stimulation, one participant had a slightly higher number of outliers of 9.6%.

After ensuring Gaussian distribution of the data by means of Kolmogorov-Smirnov goodness-of-fit test, baseline synchronization performance was analyzed by means of the tap-to-tone asynchrony as well as its standard deviation. To this end, a 4 × 2 × 2 × 2 × 2 repeated measure analysis of variance (rmANOVA) with factors *tap-position* (*P* − 4 vs. *P* − 3 vs. *P* − 2 vs. *P* − 1), *stimulation* (atDCS vs. sham), *time* (pre- vs. post-tDCS), *step-change direction* (positive vs. negative), and *type of step-change* (conscious vs. nonconscious) was applied.

In a second step, error correction was determined by calculating the difference between the asynchrony in baseline and subsequent trials. The data were analyzed by means of a 6 × 2 × 2 × 2 rmANOVA with factors *tap-position* (BL vs. *P* vs. *P* + 1 vs. *P* + 2 vs. *P* + 3 vs. *P* + 4), *stimulation* (atDCS vs. sham), *time* (pre vs. post), and *step-change direction* (positive vs. negative). Analyses were calculated separately for trials with conscious and nonconscius step-changes. Intertap intervals (ITI) were additionally calculated for pre- and postperturbation trials and analyzed by means of a 2 × 2 × 2 × 2 × 2 rmANOVA with factors *stimulation* (atDCS vs. sham), *time* (pre vs. post), *step-change direction* (positive vs. negative), *type of step-change* (conscious vs. nonconscious), and *state* (pre- vs. postperturbation trials). The participants' guesses regarding the applied stimulation condition were analyzed by means of the chi-square test. Statistics were conducted using IBM SPSS Statistics 25. *p* values below 0.05 after correction for multiple comparisons by means of the sequential Bonferroni correction [44] were considered to be significant. Greenhouse-Geisser correction was applied whenever sphericity assumption was violated.

## 3. Results

The analysis of the stimulation questionnaire suggests that the stimulation type was correctly identified in 42% of all sessions. Although a slightly higher hit-rate was found following atDCS (44.4%) as compared to sham stimulation (38.9%), the recognition rate was below chance level and did not significantly differ between stimulation conditions (*χ*^2^(1) = 0.222, *p* = 0.637). Adverse effects of tDCS like a slight tingle or burning below the stimulation electrodes were reported in 26 of 36 sessions (anodal: 13/18, sham: 13/18). One participant reported tingle of the right arm during anodal tDCS, and another one reported twitches of the right arm during sham stimulation. Further side effects were not reported.

### 3.1. Baseline Synchronization Performance

**Figure 3:**
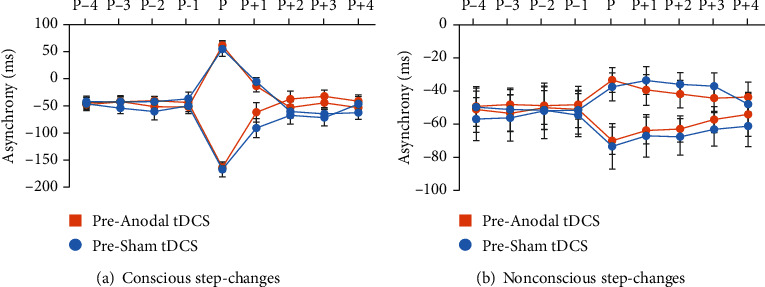
Mean tap-to-tone asynchrony in trials with conscious (a) and nonconscious (b) step-changes prior to tDCS. The analysis suggests stable synchronization performance in baseline trials (*P*–4–*P*–1). A step-change (*P*) in either direction yielded a significant deviation from the mean baseline asynchrony. Error bars delineate the standard error of the mean.

The analysis of synchronization accuracy in baseline trials (i.e., the four taps preceding the step-change) suggests the well-known negative asynchrony indicating the tap leading the tone (for review articles, refer to [1, 2]). ANOVA suggests a significant main effect of *time* (*F*(1, 17) = 17.262, *p* = 0.001, *η*_*p*_^2^ = 0.504) indicating larger negative asynchronies after stimulation (–67.92 ± 10.98 ms) as compared to prestimulation trials (–51.53 ± 10.15 ms) that occurred independent of stimulation condition. All other comparisons were not significant (*p* > 0.243, *η*_*p*_^2^ < .080).  Figure 3 indicates the mean negative asynchrony separately for conscious and nonconscious step-changes prior to tDCS.

The analysis of synchronization variability does neither suggest significant main effects of *stimulation* and *time* nor significant interactions including both factors (*p* > 0.094, *η*_*p*_^2^ < 0.156). The baseline synchronization performance is summarized in Table 1.

Since the analysis did not provide evidence for significant differences between tap-positions (i.e., *P* − 4, *P* − 3, *P* − 2, and *P* − 1), the data were averaged across positions, separately for each experimental condition serving as mean baseline (BL) performance for further analyses.

### 3.2. Error Correction

**Figure 4:**
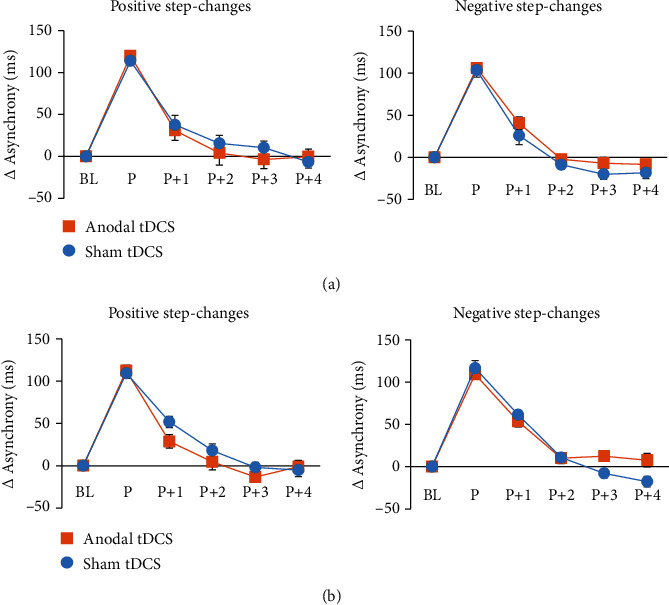
Mean asynchrony difference with respect to baseline trials in perturbation (*P*) and postperturbation (*P* + 1–*P* + 4) trials associated with conscious step-changes. (a) indicates data prior to tDCS; (b) depicts data following tDCS. The analysis suggests that independent of direction of step-changes baseline performance was achieved at *P* + 2. Anodal tDCS did not significantly modulate task performance. Error bars represent the standard error of the mean.

In a next step, differences between mean BL and tap-to-tone asynchronies in perturbation (*P*) and postperturbation trials (*P* + 1–*P* + 4), respectively, were calculated. The analysis of conscious step-changes suggests a significant main effect of *tap-position* (*F*(3.2,54.5) = 286.499, *p* < 0.001, *η*_*p*_^2^ = 0.944), indicating significantly larger differences with respect to baseline at *P* (*t*(17) = –41.718, *p* < 0.001) and *P* + 1 (*t*(17) = –9.330, *p* < 0.001). Independent of step-change direction, time point of measurement (pre vs. post tDCS), and stimulation condition baseline performance was achieved at *P* + 2 (*t*(17) = –1.707, *p* = 0.106). The data are summarized in Figure 4.

**Figure 5:**
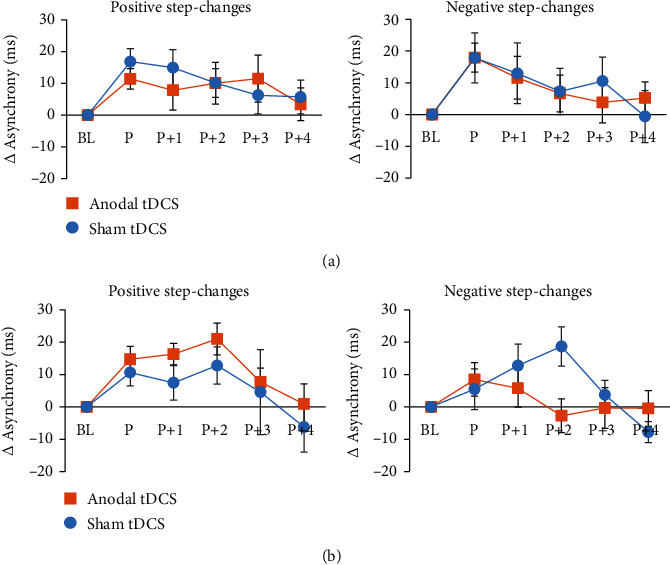
Mean asynchrony differences with respect to baseline trials in perturbation (*P*) and postperturbation (*P* + 1–*P* + 4) trials associated with nonconscious step-changes. (a) indicates data prior to tDCS; (b) depicts data following tDCS. Prior to tDCS, baseline performance was achieved at *P* + 4 independent of step-change direction. Following atDCS, the asynchrony returned to baseline at *P* + 2 in trials with negative step-changes. Error bars represent the standard error of the mean.

**Figure 6:**
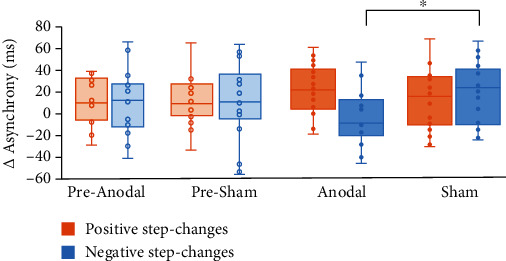
Mean asynchrony differences in nonconscious perturbation trials at *P* + 2. Following atDCS, differences were significantly smaller as compared to sham stimulation in trials with negative step-changes. Following positive step-changes, no significant differences between stimulation conditions emerged (^∗^*p* < 0.05). The horizontal line indicates the mean, and whiskers delineate minimal and maximal values.

The analysis of nonconscious perturbation trials yielded a significant *tap position x stimulation x time x step-change direction* interaction (*F*(5, 85) = 2.545, *p* = 0.034, *η*_*p*_^2^ = 0.130). Post hoc ANOVA for each position revealed significant differences at *P* + 2, only (*F*(1, 17) = 10.217, *p* = 0.005, *η*_*p*_^2^ = 0.375) suggesting significantly smaller asynchrony differences following atDCS as compared to sham stimulation (*t*(17) = –2.106, *p* = 0.050) which did not significantly differ from baseline (*t*(17) = 0.523, *p* = 0.608). The latter result indicates that in this condition, baseline performance was already achieved at *P* + 2. Prior to stimulation, the asynchronies returned to baseline level, not before *P* + 4, independent of step-change direction and stimulation condition (*p* > 0.100). The data are summarized in Figures 5 and 6.

The analysis of ITIs suggests significant differences after conscious step-changes as compared to baseline trials as indicated by a significant *type of step-change x state* interaction (*F*(1, 17) = 10.246, *p* = 0.005, *η*_*p*_^2^ = 0.376). But, this effect was neither significantly modulated by stimulation nor by time (*F*(1, 17) = 0.000, *p* = 0.996, *η*_*p*_^2^ = 0.000). The data are summarized in Tables 1 and 2.

## 4. Discussion

The present study is aimed at investigating whether atDCS applied to the left M1 modulates correction of conscious and nonconscious timing errors of the contralateral right hand. The data suggest a distinct facilitating effect of atDCS on error correction following nonconscious negative step-changes. More precisely, atDCS was associated with a faster return to baseline performance as compared to sham stimulation in this condition, only. Noteworthy, synchronization performance in baseline trials prior to step-changes was not affected by atDCS, replicating a previous finding [33]. All in all, the data suggest that the correction of externally induced timing errors requires different mechanisms than the correction of internal timing errors observed during synchronization with respect to a regular pacing signal.

### 4.1. Baseline Performance

The analysis of baseline synchronization performance suggests the well-known negative asynchrony associated with synchronization in the subsecond range (for review articles, refer to [1, 2]). The comparison between pre- and poststimulation trials suggests larger mean asynchronies after tDCS. Importantly, this effect occurred independent of stimulation type and might therefore indicate reduced task-related attention associated with progression of experimental time. The data suggest that atDCS applied to M1 does not significantly modulate synchronization performance replicating previous findings [33]. Smaller tap-to-tone asynchronies following 1 Hz repetitive TMS (rTMS) of M1 were found in a previous study [36], but this finding was not replicated by applying cathodal tDCS to M1 [33]. The data point to the hypothesis that rTMS and tDCS may have distinct effects on M1 excitability yielding different behavioral effects.

### 4.2. Error Correction

Prior to tDCS, faster error correction of conscious as compared to nonconscious step-changes was found replicating previous findings. In line with previous data [3, 4, 45], baseline performance following conscious deviations was achieved already at the second tap after the perturbation (*P* + 2) and remained on this level. In contrast to this, following nonconscious step-changes, the asynchrony returned to baseline not before the fourth tap (*P* + 4). Previous studies suggest faster correction for positive as compared to negative deviations [3, 4, 45, 46]. In contrast to this, the present data do not provide evidence for significant differences depending on the direction of perturbation replicating previous findings [47, 48].

The involvement of the cerebellum [3], the left PMC [45], and the dorsolateral prefrontal cortex in conscious motor control and error correction [5] has been suggested, while ventral prefrontal areas may be stronger involved in nonconscious motor adaptation [5]. The present data suggest M1 as part of a network distinctively subserving nonconscious error correction. Bijsterbosch and colleagues used functional magnetic resonance imaging (fMRI) to characterize the brain networks associated with sensorimotor synchronization and nonconscious error correction and did not find differences between brain activation patterns [3]. In contrast to this result, the present data suggest that atDCS of M1 distinctively modulates nonconscious error correction leaving synchronization performance in the subsecond range unaffected. The data are in line with the hypothesis that external timing perturbations require different mechanisms than internal timing errors observed during synchronization with respect to a regular pacing signal (for a review article, refer to [2]). We realize that this result contradicts the findings by Doumas et al. showing a significant modulation of the mean negative asynchrony while no effect on error correction was obtained [36]. Importantly, in that study, 1 Hz rTMS was applied to M1, while in the present study, atDCS was used. Although speculative, it might be possible that correction of nonconscious errors might be more susceptible to increased M1 excitability than to its reduction.

The present data point towards a facilitating effect of anodal M1 tDCS on nonconscious error correction following negative but not positive step-changes. Tapping in synchrony with an isochronous metronome is associated with a mean negative asynchrony as indicated by the tap leading the tone by several tens of milliseconds (for review articles, refer to [1, 2]). Although the causes for this tendency are still not fully understood, participants are more sensitive in recognizing and correcting positive than negative asynchronies pointing to asymmetrical error tolerance (for a review article, refer to [49]). Support for this hypothesis comes from an electroencephalography (EEG) study showing the involvement of a fronto-parietal network for the correction of positive but not negative liminal deviations [21]. Those data suggest an inhibitory influence of fronto-medial areas on M1 contralateral to the moving hand yielding lengthening of the tap interval. Anodal tDCS applied to M1 has been shown to increase its excitability (e.g., [26, 50]). Therefore, shortening of the tap interval may occur facilitating detection and correction of negative step-changes. Noteworthy, this presumed effect did not modulate the tapping speed as indicated by the nonsignificant effect of atDCS on intertap intervals during baseline synchronization.

The data do not provide evidence for facilitation of conscious error correction following atDCS. Conscious error correction has been shown to be more susceptible to reduced PMC excitability rather than to its increase as suggested by means of theta-burst stimulation [45]. Although we cannot rule out that in the present study cathodal tDCS may have affected conscious error correction, findings from a previous study applying 1 Hz rTMS to M1 argue against this hypothesis [36]. Those data as well as the present findings indicate that M1 is not part of a network supporting conscious error correction. All in all, evidence exists that conscious and liminal error correction might be due to voluntary control [51] associated with a network comprising premotor and prefrontal areas as well as the cerebellum [3, 5, 7, 21, 45]. The present findings support the hypothesis of an “automatic” timing system associated with nonconscious error correction being linked to motor circuits [12].

### 4.3. Limitations

The most critical issue associated with tDCS is its low spatial specificity (for review articles, refer to [31, 39, 40, 52, 53]) in particular when a bipolar electrode montage is used as in the present study [54]. Besides spreading of the electrical field (e.g., [55]), stimulation effects on remote brain areas due to functional connectivity (e.g., [56]; for review articles, refer to [24, 30, 31, 40]) have been shown. It has been argued that the transmission time from the auditory cortex to premotor and motor cortices might be too slow to drive error correction supporting the significance of a subcortical pathway (for a review article, refer to [57]). Hence, we here would not attribute the stimulation effect observed in the present data exclusively to M1. Rather we would argue that M1 opens the opportunity to interact with a brain network associated with nonconscious error correction in the temporal domain. Due to the exploratory nature of the study, we desisted from adopting a control site for tDCS application. We can therefore not exclude the possibility that stimulation of other brain areas may have yielded comparable results [53]. This hypothesis needs to be tested in future studies.

Noteworthy, the exact stimulation effects depend on the strength of the electrical field and the conductivity of the stimulated tissue as well as the orientation of pyramidal neurons in the stimulated area with respect to the electrical field induced by tDCS contributing to interindividual differences [27, 54] (for review articles, refer to [22, 31, 39, 40, 53]). In addition, a recent study suggests that the TMS hotspot differs from fMRI activation associated with finger tapping [58] raising the question whether the stimulation area chosen in the present study was optimal. Despite this weakness, it has been shown that the chosen electrode montage yields significant modulation of M1 excitability (e.g., [26, 50]). Another crucial issue is the relatively low stimulation intensity of 250 *μ*A. But, excitability after-effects of M1-tDCS do not linearly correlate with stimulation intensity. Rather, lower intensities (i.e., 0.5–1.0 mA) yielded comparable effects of anodal tDCS on M1 excitability as higher intensities (i.e., 1.5–2.0 mA) [50]. Noteworthy, in that study, a larger electrode covering M1 was chosen than in the present study yielding current densities below the active electrode between 0.014 and 0.028 mA/cm^2^ in the lower intensity stimulation conditions corresponding to the intensity applied in the present study. Another critical issue is that, step-changes occurred on the same position suggesting that the participants were able to predict the next step-change. Although we cannot exclude an effect of predictability, it cannot explain the observed stimulation effect since a comparable effect should occur independent of perturbation direction. Finally, the instruction highlighting the possibility of temporal irregularities may have cued to particularly attend potential step-changes. Therefore, trials with 2% step-changes may have been under the guidance of an explicit error-correction network. Although we cannot rule out an effect of the instruction, the distinct effect on the correction of negative nonconscious step-changes argues against this possibility.

## 5. Conclusions

The present study suggests M1 as important node within a network subserving nonconscious error correction of negative step-changes in the temporal domain going beyond sensorimotor synchronization.

## Figures and Tables

**Table 1:** Mean baseline synchronization performance (± standard error of the mean).

	Nonconscious		Conscious	
	Positive	Negative	Positive	Negative
Presham				
Asynchrony	–48.4 ± 10.2 ms	–46.8 ± 11.9 ms	–53.3 ± 12.3	–41.0 ± 10.3 ms
Variability	35.3 ± 4.0 ms	34.2 ± 2.2 ms	31.9 ± 2.9 ms	32.0 ± 2.4 ms
ITI	901.6 ± 2.1 ms	900.2 ± 2.0 ms	897.8 ± 1.4 ms	899.7 ± 1.8 ms
Sham				
Asynchrony	–75.6 ± 13.9 ms	–73.4 ± 13.6 ms	–73.8 ± 13.5 ms	–71.4 ± 15.1 ms
Variability	42.2 ± 5.1 ms	34.5 ± 3.6 ms	32.3 ± 2.8 ms	36.5 ± 4.0 ms
ITI	900.0 ± 1.6 ms	896.1 ± 1.6 ms	882.7 ± 2.0 ms	901.0 ± 1.2 ms
Pre-atDCS				
Asynchrony	–51.5 ± 9.7 ms	–44.8 ± 8.2 ms	–43.3 ± 7.2 ms	–47.53 ± 8.6 ms
Variability	31.0 ± 2.3 ms	32.7 ± 2.3 ms	34.5 ± 2.5 ms	28.3 ± 2.3 ms
ITI	899.5 ± 1.1 ms	898.9 ± 1.0 ms	898.5 ± 1.0 ms	899.0 ± 2.0 ms
atDCS				
Asynchrony	–55.9 ± 10.7 ms	–57.7 ± 8.7 ms	–63.8 ± 12.4 ms	–71.6 ± 13.6 ms
Variability	32.2 ± 2.9	34.3 ± 4.1 ms	33.5 ± 3.4 ms	33.7 ± 3.5 ms
ITI	900.8 ± 1.9 ms	898.7 ± 1.4 ms	898.9 ± 1.5 ms	894.5 ± 1.5 ms

**Table 2:** Mean intertap interval after step-changes (± standard error of the mean).

	Nonconscious		Conscious	
	Positive	Negative	Positive	Negative
Presham	916.7 ± 2.3 ms	880.2 ± 1.9 ms	1040.1 ± 3.0 ms	760.6 ± 2.2 ms
Sham	920.2 ± 1.9 ms	882.7 ± 2.1 ms	1040.5 ± 2.4 ms	762.2 ± 2.4 ms
Pre-atDCS	915.4 ± 2.2 ms	880.2 ± 1.4 ms	1041.6 ± 2.8 ms	760.2 ± 2.1 ms
atDCS	915.6 ± 2.1 ms	881.0 ± 1.2 ms	1040.4 ± 2.8 ms	770.6 ± 4.2 ms

## Data Availability

Anonymized raw data are available upon request. To this end, please contact the corresponding author.
